# Rice *Snl6,* a Cinnamoyl-CoA Reductase-Like Gene Family Member, Is Required for NH1-Mediated Immunity to *Xanthomonas oryzae* pv. *oryzae*

**DOI:** 10.1371/journal.pgen.1001123

**Published:** 2010-09-16

**Authors:** Rebecca S. Bart, Mawsheng Chern, Miguel E. Vega-Sánchez, Patrick Canlas, Pamela C. Ronald

**Affiliations:** 1 Department of Plant Pathology, University of California Davis, Davis, California, United States of America; 2 Joint Bioenergy Institute, Emeryville, California, United States of America; The University of North Carolina at Chapel Hill, United States of America

## Abstract

Rice NH1 (NPR1 homolog 1) is a key mediator of innate immunity. In both plants and animals, the innate immune response is often accompanied by rapid cell death at the site of pathogen infection. Over-expression of NH1 in rice results in resistance to the bacterial pathogen, *Xanthomonas oryzae* pv. *oryzae* (*Xoo*), constitutive expression of defense related genes and enhanced benzothiadiazole (BTH)- mediated cell death. Here we describe a forward genetic screen that identified a suppressor of NH1-mediated lesion formation and resistance, *snl6*. Comparative genome hybridization and fine mapping rapidly identified the genomic location of the *Snl6* gene. *Snl6* is a member of the cinnamoyl-CoA reductase (CCR)-like gene family. We show that *Snl6* is required for NH1-mediated resistance to *Xoo*. Further, we show that *Snl6* is required for pathogenesis-related gene expression. In contrast to previously described CCR family members, disruption of *Snl6* does not result in an obvious morphologic phenotype. *Snl6* mutants have reduced lignin content and increased sugar extractability, an important trait for the production of cellulosic biofuels. These results suggest the existence of a conserved group of CCR-like genes involved in the defense response, and with the potential to alter lignin content without affecting development.

## Author Summary

Plants possess potent and effective endogenous methods for responding to pathogen attacks, referred to as plant innate immunity. In this report we further our understanding of rice innate immunity through characterization of the *Snl6* gene. The *snl6* mutant was identified from a mutant screen for positive regulators of immunity. While innate immunity represents a powerful agronomic tool, identification of desirable genes from crop species is limited by the slow and laborious nature of map-based cloning. Here we describe our methodology of combining comparative genome hybridization and fine mapping to rapidly identify the *Snl6* gene. *Snl6* is distantly related to members of the cinnamoyl-CoA reductase gene family, is required for pathogenesis gene expression and resistance to the bacterial pathogen *Xanthomonas oryzae* pv. *oryzae*. Snl6 mutants have reduced lignin content and increased sugar extractability, an important trait for the production of cellulosic biofuels.

## Introduction

Plant innate immunity is governed by a complex signaling network [1]. Hallmark events during the immune response include localized cell death at the site of pathogen recognition (known as the hypersensitive response (HR)), release of reactive oxygen species (ROS), pathogen related (PR) gene induction, callose deposition and cell wall lignification [2], [3], [4]. The HR, ROS and PR gene production are thought to be physically harmful towards the invading pathogen while callose and lignin deposition create a physical barrier to limit pathogen entry and spread [4], [5], [6], [7], [8].

NPR1 (Non-expressor of PR genes-1) is a central regulator of the disease response in *Arabidopsis* and has been studied extensively [9], [10], [11], [12], [13]. Plants deficient in NPR1 expression lack PR gene accumulation after pathogen treatment, display increased susceptibility to pathogens and fail to initiate systemic acquired resistance (SAR) [11]. Over-expression of the rice NPR1 ortholog, NH1 (NPR1 homologue 1) (NH1ox) in rice results in enhanced resistance to the bacterial pathogen *Xanthomonas oryzae* pv. *oryzae* (*Xoo*), constitutive expression of PR genes and a BTH (benzothiadiazole)-mediated cell death phenotype [14]. Enhanced cell death (also called lesion mimic phenotypes) has been correlated with resistance [15], [16].

Here we describe a mutant screen to identify additional components of the rice innate immune response. NH1ox seeds (M_0_) were treated with fast neutron mutagenesis to generate an M_1_ population segregating for genomic deletions. Sixty thousand M_1_ lines were screened for alterations in the immune response after treatment with BTH. Plants that did not develop BTH-induced lesions were collected as putative suppressors of NH1-mediated lesion formation (*snl*) mutants. In this report we focus on one mutant, *snl6*. To expedite the cloning of *Snl6* we employed comparative genome hybridization (CGH) [17], [18], [19], [20], [21], [22], [23], [24] to identify five deletions, ranging in size from 3 kb to 100.5 kb, in the *snl6* mutant line. Only the deletion on rice chromosome 1 segregated with the *snl6* mutant phenotype. Subsequent RNAi and allelic complementation confirmed that *Snl6* encodes a member of the cinnamoyl-CoA reductase-like gene family. *snl6* mutant lines fail to develop the NH1-mediated lesions, PR gene activation and resistance phenotypes. Further we show that *Snl6* is not required for resistance mediated by the rice pattern recognition receptor, Xa21 [25], [26]. Finally, *snl6* mutant lines show decreased phloroglucinol staining without an obvious morphologic phenotype.

## Results

### Identification of the *Snl6* Mutant That Suppresses NH1-Mediated Lesion Formation

**Figure 1:**
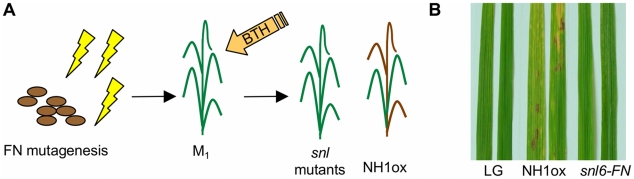
Identification of the *snl6-FN* mutant. (**A**) Cartoon of mutant screen used to identify suppressors of BTH-induced, NH1-mediated lesion mimic (*snl*) mutants. NH1ox seeds were treated with Fast Neutron (FN) mutagenesis (M_1_). M_2_ plants were treated with BTH and then screened for the appearance of the lesion mimic phenotype. *snl6-FN* (line 11-3) did not develop lesions after BTH treatment. (**B**) 8-week old plants were treated with BTH. Plants were assessed for the presence of the lesion mimic phenotype. Image was taken two weeks after BTH treatment. LG, NH1ox and *snl6-FN* (line: 11-3-2) were compared.

We have previously reported that over expression of the rice NPR1 homologue, NH1, in a LiaoGeng (LG) background, confers resistance to the bacterial pathogen *Xanthomonas oryzae* pv. *oryzae* (*Xoo*) and leads to a spontaneous cell death phenotype [14]. The cell death phenotype is enhanced by application of the salicyclic acid functional analog BTH [27]. We designed a screen to identify mutants incapable of developing this cell death phenotype (Figure 1A). NH1ox seeds were treated with fast neutron (FN) mutagenesis (see *Materials and Methods*). M_1_ progeny from 4,000 M_0_ were harvested in the rice field. Approximately 60,000 M_1_ individuals were grown in the rice field and sprayed with 10 mM BTH at 5 weeks and 7 weeks and scored for cell death at 10 weeks. BTH treatment of NH1ox plants in the field resulted in cell death, visualized by discrete lesions on the leaves. While most plants showed the typical NH1ox cell death phenotype, approximately 20 plants showed little to no cell death and were therefore named suppressor of BTH-induced, NH1-mediated lesion mimic (*snl*) mutants. The suppressed cell death phenotype of *snl6-FN* (fast neutron allele) was confirmed in the greenhouse (Figure 1B).

**Figure 2:**
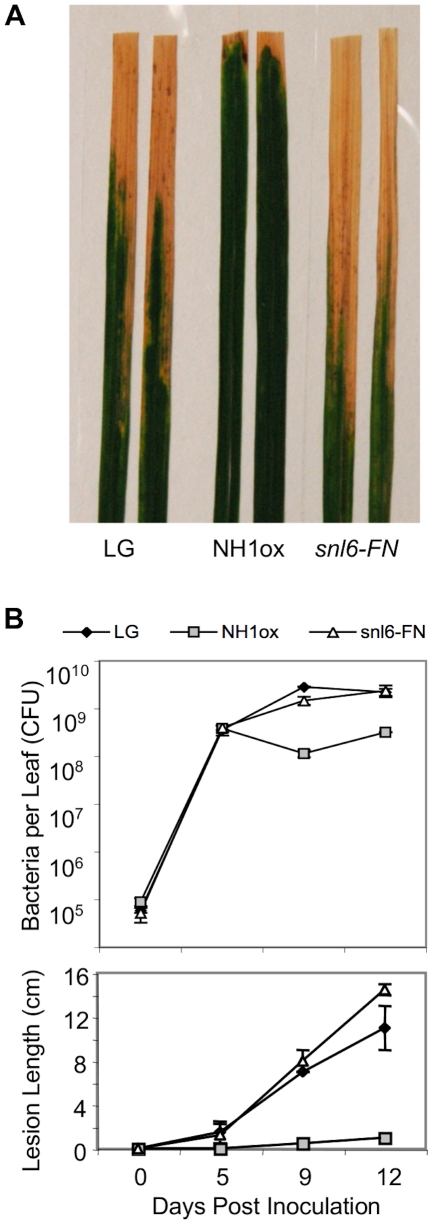
*Snl6* is required for NH1ox-mediated resistance to *Xoo*. 8-week old plants were challenged with *Xoo*. (**A**) Lesion length development after 12 days. (**B**) Total bacterial populations per leaf (top) and lesion lengths (bottom) were measured at 0, 5, 9 and 12 days post inoculation. Mean, ± range (n = 2), are displayed. The growth curve experiment was repeated twice with similar results. LG, NH1ox and *snl6-FN* (line: 11-3-2) were compared.

As lesion mimic mutants often have an accompanying resistance phenotype [15], [16] we hypothesized that *snl6-FN* plants may show enhanced susceptibility to *Xoo.* Indeed, *snl6-FN* lines show longer water-soaked lesions after infection with *Xoo* and support greater populations of *Xoo* cells than NH1ox plants (Figure 2A and 2B), indicating that *Snl6* is required for NH1-mediated resistance. To ensure that the *snl6-FN* phenotype did not represent a mutation in the NH1 over-expression transgene, we confirmed the over-expression of *NH1* in the *snl6-FN* line (Figure S1).

### Comparative Genome Hybridization Combined with Fine Mapping Locate *Snl6* on Rice Chromosome 1

**Figure 3:**
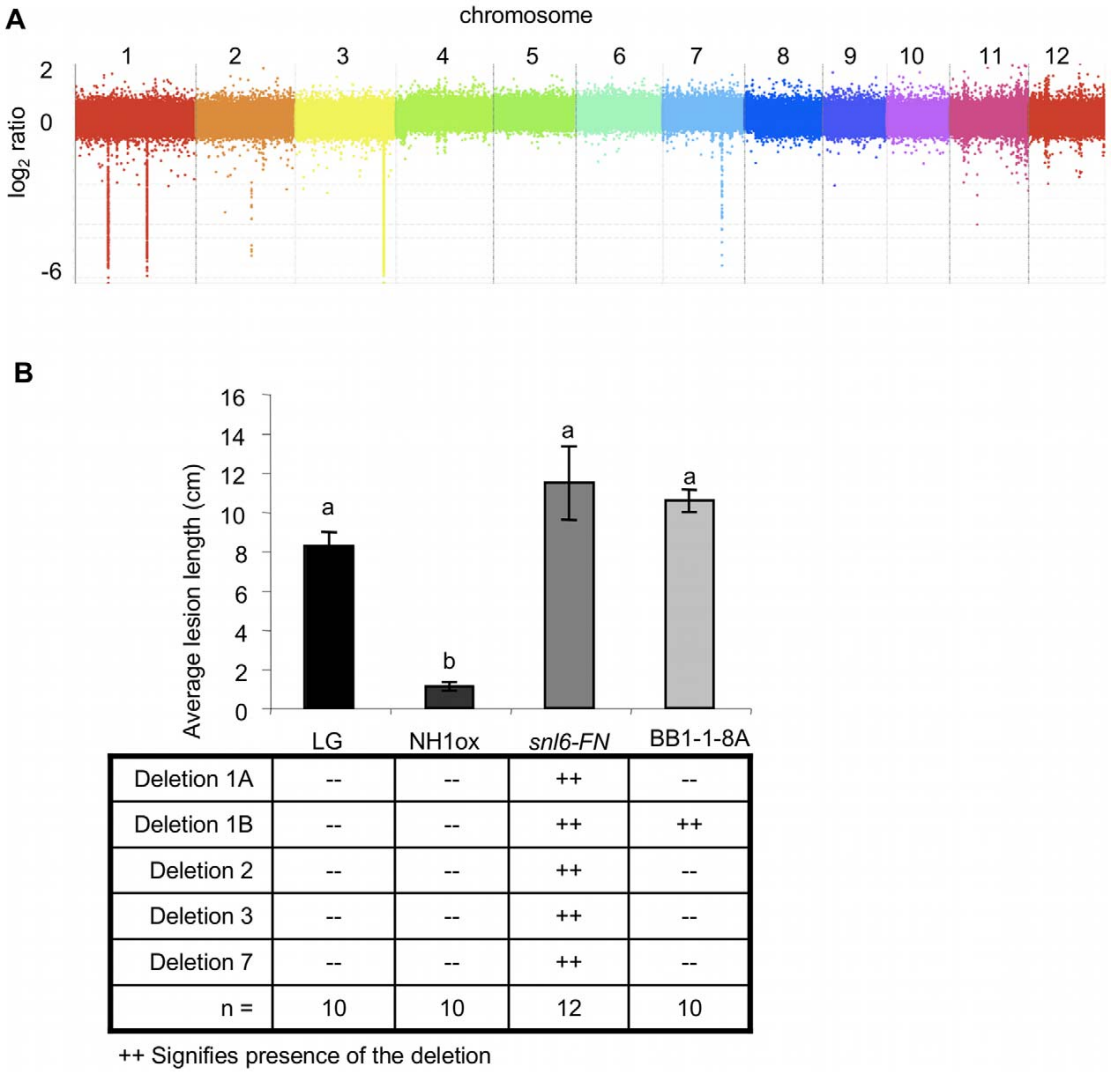
*Snl6* segregates with deletion 1B. (**A**) Comparative Genome Hybridization (CGH) between NH1ox and *snl6-FN* (line: 11-3-2). Each spot represents an average of all probes in a 1.5 kb region. Identified deletions were named based on chromosome number as follows Deletion 1A, 1B, 2, 3, 7. (**B**) Plants were scored for resistance after challenge with *Xoo*. mean ± s.e.m, Different letters represent significant difference (p<0.05). LG, NH1ox, *snl6-FN* (line 11-3-2), BB1-1-8A (F2 mapping population individual) progeny.

Conventional map-based cloning, while effective, is slow and laborious [25], [28]. With the availability of fully sequenced genomes, an alternative method for gene cloning has emerged called comparative genome hybridization (CGH) [29]. CGH uses tiling arrays to physically compare two genomes. In CGH, genomic DNA from each plant is fragmented and differentially labeled. The labeled DNA is then hybridized to a tiling array, composed of probes where each probe corresponds to a known location on the reference genome. Probes that show strong hybridization with the parent but not the mutant, indicate deleted regions on the mutant genome. Because the *snl6-FN* mutant was created with fast neutron mutagenesis, which generally induces deletions on chromosomes [30], we employed CGH to expedite the cloning of *Snl6*. In collaboration with Nimblegen, we designed a full genome tiling array for rice (japonica cultivar) with an average probe spacing of one 50-mer probe every 146 bp. NH1ox and *snl6-FN* genomic DNA was prepared, fragmented and labeled with Cy3 or Cy5, respectively. Relative hybridization intensities for each probe are reported as: log_2_(*snl6-FN*/NH1ox). A negative log_2_ ratio represents a deletion in the genome of the *snl6-FN* mutant. CGH revealed 5 deletions (Figure 3A), encompassing 35 annotated genes (Table S1), in the genome of *snl6-FN* and each was confirmed through PCR.

Next, we created an F2 mapping population (line: BB1-1) and individuals were genotyped for the NH1ox transgene, each deletion and then scored for resistance to *Xoo*. Only the second deletion on chromosome 1 (Deletion 1B) cosegregated completely with susceptibility. One individual showed a recombination event between the two deletions on chromosome 1. We analyzed the next generation of this line (BB1-1-8A) and confirmed the susceptibility (Figure 3B). Deletion 1B contains 3 annotated, non-transposon genes: LOC_Os01g45160, LOC_OS01g45190 and LOC_Os01g45200 (Table S1). The former does not have any associated expression data (based on EST libraries and publicly available microarray data) and thus we de-prioritized this gene as an unlikely candidate for *Snl6*. LOC_Os01g45200 is annotated as a cinnamoyl CoA-reductase (CCR)-like gene (TIGR v6.1). Because CCRs catalyze the first committed step in the lignin biosynthetic pathway and have previously been linked to the defense response [31], [32], LOC_Os01g45200 became our top candidate for *Snl6*.

### *Snl6* Encodes a Member of the Cinnamoyl CoA-Reductase (CCR)-Like Gene Family

**Figure 4:**
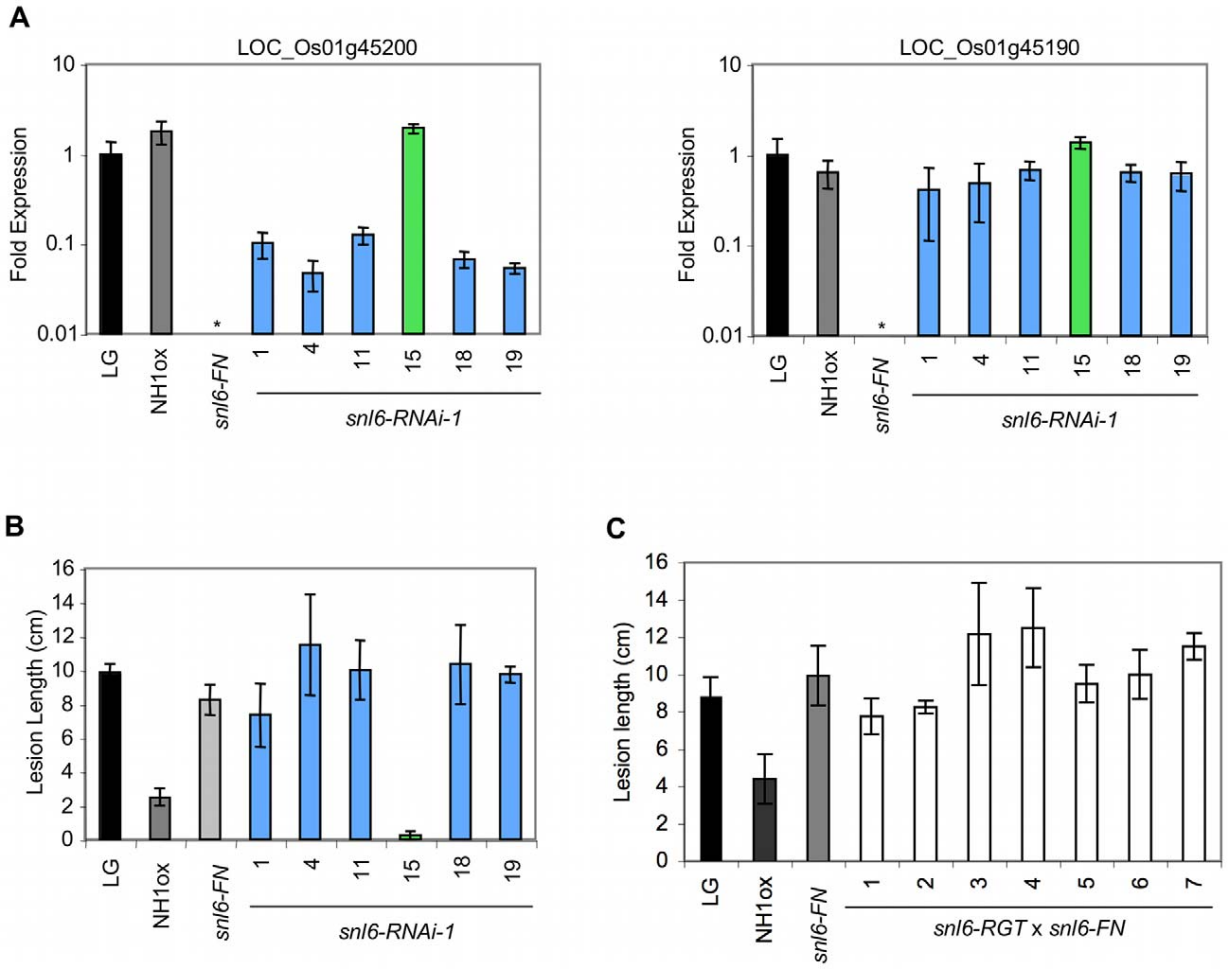
RNAi and allelic complementation for *Snl6*. (**A**) Expression of LOC_Os01g45190 and LOC_Os01g45200 in *snl6-RNAi-1* progeny, ± s.d., n = 3 (**B**) Lesion lengths of *snl6-RNAi-1* T1 progeny, 14 days after inoculation with *Xoo.* ± s.d., n = 3; * =  expression level below background (**C**) Allelic complementation test. Lesion lengths 11 days after challenge with *Xoo*. ± s.d., n = 3. LG, NH1ox, *snl6-FN* (line 11-3-2), *snl6-RNAi-1* progeny (T_1_), *snl6-RGT* (RGT6140B_5.1) x *snl6-FN* (line: 11-3-2) F1 individuals.

To determine if LOC_Os01g45200 is *Snl6*, we generated transgenic plants expressing an inverted repeat RNAi construct (*snl6-RNAi*) to silence LOC_Os01g45200 in an NH1ox background (Figure S2). *snl6-RNAi* targets 425 bp of the 3′ end of LOC_Os01g45200. This region is 74% identical to the closest homolog, LOC_Os05g50250, however this gene is intact in the *snl6-FN* and *snl6-RGT* (described below) alleles. Consequently we know that LOC_Os05g50250 does not contribute to the *snl6* mutant phenotype. Four independent RNAi lines (primary transgenics, T0) challenged with *Xoo* displayed enhanced susceptibility as compared to the NH1ox control (Figure S3). Among six T1 progeny of *snl6-RNAi-1*, the enhanced susceptibility phenotype segregated perfectly with the presence of the transgene and with reduced expression of LOC_Os01g45200 (Figure 4A and 4B). LOC_Os01g45190 showed wild-type expression levels in all *snl6-RNAi-1* progeny indicating that this gene does not contribute to the *snl6* susceptibility phenotype. While *snl6-RNAi-1* shows higher expression of *Snl6* than the deletion allele, *snl6-FN*, the resulting lesion lengths for these different lines are comparable suggesting that *Snl6* may not function in a dosage dependent manner. Next, we identified an insertion line from the Rice Transposon Flanking Sequence Tag (FST) Database [33]. We designated this line, *snl6-RGT* (Figure S2) and performed an allelic complementation test by crossing *snl6-RGT* with *snl6-FN* (recessive) and analyzing the progeny. Successful crosses were confirmed through PCR. All seven F1 individuals challenged with *Xoo* showed levels of enhanced susceptibility similar to that of *snl6-FN* (Figure 4C). These lines carry one copy of the NH1ox transgene, which we have previously shown is sufficient to confer high levels of resistance to *Xoo* [14]. Thus we conclude that *snl6-RGT* and *snl6-FN* are allelic. Taken together these data confirm that LOC_Os01g45200 is *Snl6*, a cinnamoyl-CoA reductase (CCR)-like gene.

*Snl6* encodes a predicted protein of 364 amino acids (39.5 kDa). CCRs exist as multi-gene families with at least 7 and 14 annotated members in Arabidopsis and rice, respectively. Previously described CCR genes show limited identity with *Snl6* [31], [32], [34], [35] (Figure S4). The closest predicted rice paralog is LOC_Os05g50250, sharing 73% identity at the amino acid level. Sorghum, *Brachypodium* and maize all have predicted orthologs (EES03334.1, 2g44800.1 and ACR34585.1 with 86%, 81% and 82%, amino acid identity, respectively). The closest predicted ortholog in *Arabidopsis thaliana* is AT5G14700 with 44% amino acid identity. None of these closest predicted orthologs have been functionally characterized. In Arabidopsis, the distantly related genes, AtCCR1 (25% identity) and AtCCR2 (27% identity) have been attributed a primary role in development or pathogen attack, respectively. They appear to be able to partially compensate for each other [32].

### *Snl6* Is Not Required for XA21-Mediated Resistance

**Figure 5:**
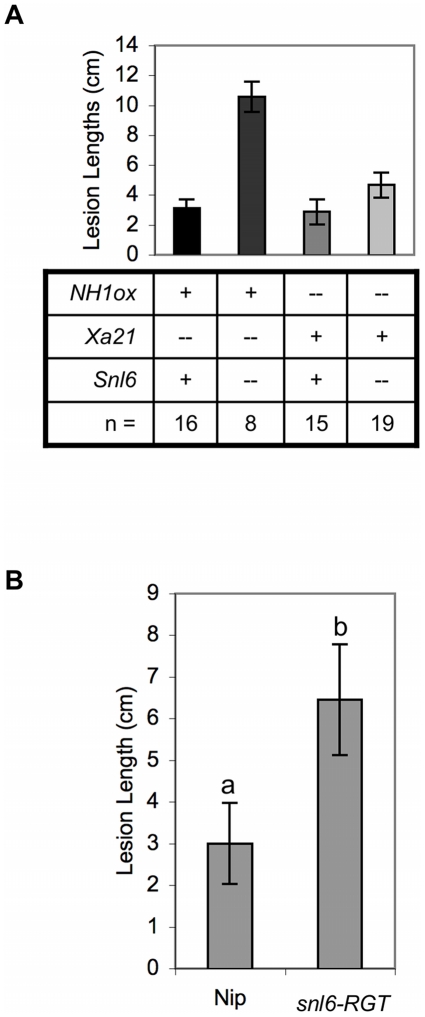
*Snl6* contributes to resistance in the absence of NH1ox. (**A**) Individuals from line BB1-1 (*snl6-FN* (line:11-3-2) x Ubi-Xa21-kitaake (line 7A-8)) were genotyped for the presence of *NH1ox*, *Xa21* and *Snl6*. 8-week old plants were moved to the growth chamber and challenged with *Xoo*. Lesion lengths were measured after 12 days. Mean ± s.e.m. and number of individuals (n) are reported. (**B**) Average lesion lengths 11 days after *Xoo* inoculation. Nip (wildtype cultivar nipponbare); *snl6-RGT* (T-DNA insertion in *Snl6*). ± s.e.m., n = 12 (nip) or 18 (*snl6-RGT*). Letters (a, b) indicate significant difference (p<0.05).

Both NH1ox and XA21-mediated resistance are regulated by NRR (negative regulator of resistance) suggesting a possible overlap of these pathways [36]. To determine if *Snl6* is required for resistance mediated by XA21, we examined individual F2 progeny derived from a cross between *snl6-FN* line and a transgenic line expressing *Xa21* under control of the ubiquitin promoter [37]. These lines were segregating for *NH1ox*, *Xa21* and *Snl6*. Plants containing *Xa21* were resistant to *Xoo* independent of the presence of *Snl6* (Figure 5A). Thus we conclude that *Snl6* is required for NH1-mediated resistance, but not XA21-mediated resistance in these lines. Our results are consistent with studies from Arabidopsis showing that the PRR, FLS2, does not require NPR1 to initiate an immune response [38].

### *Snl6* Contributes to Resistance in the Absence of NH1ox

We have shown that *Snl6* contributes to NH1ox–mediated resistance. To further investigate the role of *Snl6* in the absence of NH1ox we compared inoculation data for *snl6-RGT* (lacking the NH1ox transgene) and in the genetic background of cultivar, Nipponbare used as a recipient in the transformation studies. While both lines are susceptible to inoculation with *Xoo*, *snl6-RGT* plants developed longer lesions suggesting that *Snl6* contributes to resistance in Nipponbare (Figure 5B).

### *Snl6* is Required for NH1-Mediated PR10 Gene Expression

**Figure 6:**
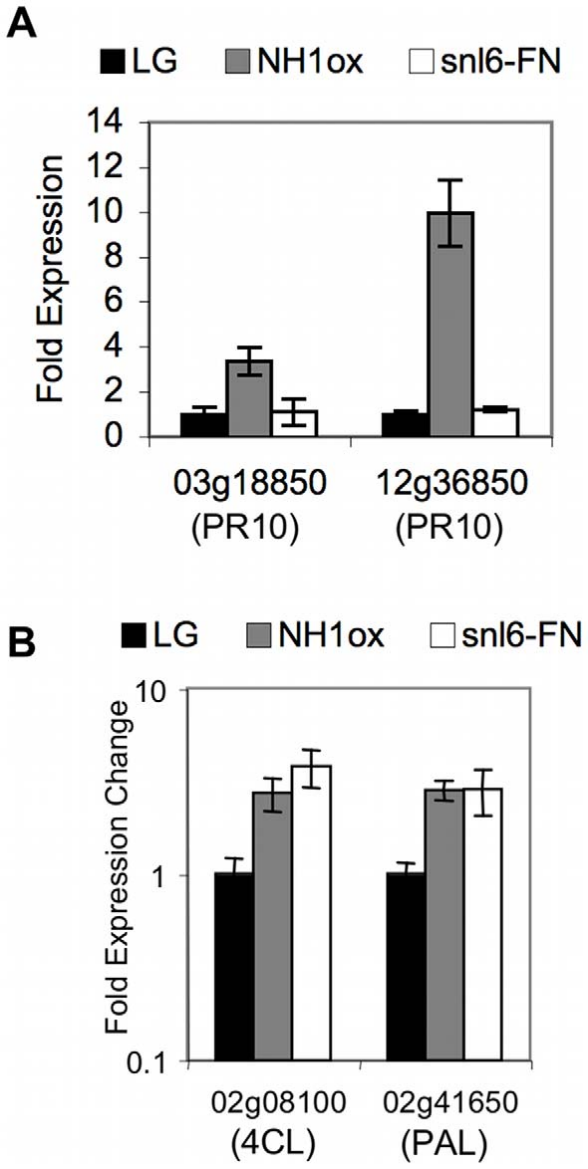
Characterization of the role of *Snl6* in the defense response. Relative gene expression levels in LG, NH1ox and *snl6-FN* (line: 11-3-2-1) lines using realtime quantitative RT-PCR, (**A**) two PR10 genes (**B**) 4CL and PAL; mean ± s.e.m, n = 3.

The NH1ox resistance phenotype is correlated with constitutively high expression levels of several PR genes [14]. To further elucidate the role of *Snl6* in innate immunity, we examined the relative expression levels of two PR10/PBZ family members (LOC_Os03g18850 and LOC_Os12g36850), in LG, NH1ox and *snl6-FN* lines. Both PR10 family members show significantly higher expression levels in NH1ox lines as compared to LG. *snl6-FN* lines while still over-expressing NH1 (Figure S1), do not show enhanced PR10/PBZ gene expression (Figure 6A). A similar result was observed for the *snl6-RNAi-1* line.

### PAL and 4-coumarate-CoA Ligase 1 (4CL) Are Co-Expressed with *Snl6*

In addition to induced PR gene expression, over expression of NH1 results in constitutive activation of phenylalanine ammonia-lyase (PAL). PAL catalyzes the first step of the highly branched phenylpropanoid pathway and has been studied for its role in lignin biosynthesis as well as defense related molecules such as salicylic acid. By analyzing publicly available microarray data we found that PAL as well as a member of the lignin specific branch of the phenylpropanoid biosynthetic pathway, 4-coumarate-CoA ligase 1 (4CL), were highly co-expressed (cc >0.6) with *Snl6.* We hypothesized that *snl6* mutant lines might contain alterations in the phenylpropanoid pathway. Both PAL and 4CL showed more than 2 fold higher expression in NH1ox and *snl6-FN* as compared to LG (Figure 6B). These results suggest that lignin biosynthesis is induced by over expression of NH1 and further suggest that *Snl6* may function downstream of PAL and 4CL, which would be consistent with the published placement of CCR in the phenylpropanoid pathway.

### *Snl6* Mutants Have Reduced Lignin and Enhanced Sugar Extractability

**Figure 7:**
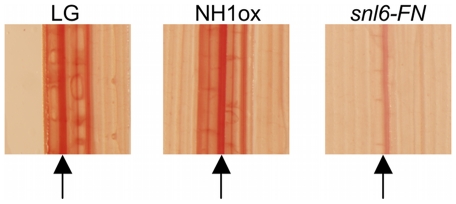
Characterization of the role of *Snl6* in the phenylpropanoid pathway. Tissue from 8-week old plants was cleared with lactic acid/phenol and stained with phloroglucinol/HCl. Images were taken at 2X magnification. Arrows point to the midrib of each sample. Experiment was repeated 5 times with similar results. Note: LG sample ripped during sample preparation.

To further elucidate the function of *Snl6*, we used the Wiesner Test (Phloroglucinol/HCl). Phloroglucinol/HCl reacts with aromatic aldehydes to produce a pink/red color and is commonly used to detect lignin [34], [39], [40]. NH1ox and LG showed clear staining, most strongly at the midvein (arrow). While some staining is observed in *snl6-FN* leaves, total staining is reduced especially at the midvein (Figure 7). Among five independent experiments, the amount of staining observed in LG leaves varied, but was consistently greater than that seen for *snl6* mutant line suggesting that additional environmental factors contribute to wildtype lignin accumulation. Notably, most previously characterized lignin mutants express a severe developmental phenotype [34], [40], [41], [42]. In contrast, no significant difference was found between *snl6-FN* and wild-type plants for plant height, seed set, tiller number or general appearance (Figure S5).

Decreased expression of CCR-like genes has been correlated with increased sugar extractability from cell walls in alfalfa and poplar, an important trait for the production of cellulosic biofuels [34], [39]. We hypothesized that this correlation may also be present in *snl6-FN* plants. We first determined that the *snl6* mutation does not affect the relative cell wall monosaccharide composition in leaves (Figure S6A). None of the individual sugars tested show a significant difference between the different genotypes (p<0.05). Next, we quantified the total sugar release after hot water pre-treatment. Compared to the LG and NH1ox controls, *snl6-FN* plants showed an increase in sugar release (p<0.05) (Figure S6B). These results are consistent with previous reports that correlate decreased lignin content with increased sugar extractability [43].

## Discussion

The ability of a plant to recognize the presence of a pathogen and mount an effective immune response is fundamental to survival. The defense response must be tightly regulated as each of these responses present a potential fitness cost to the host. The result is a complex network of signaling cascades that govern plant innate immunity. *Arabidopsis* NPR1 and rice NH1 are central regulators of plant innate immunity. Our objective in the current study was to identify additional components of the rice innate immune response thus broadening our understanding of this important biological process.

In this report, we describe the identification and characterization of rice *Snl6*. We identified *Snl6* from a screen for mutants that suppress the NH1-mediated lesion mimic phenotype. Notably, while many negative regulators of innate immunity have been identified [36], [44], [45], [46], relatively few positive regulators from rice have been characterized to date. By conducting the mutagenesis in an NH1ox background and then screening for suppressors of the lesion mimic phenotype, we were able to specifically target positive regulators, that is, genes that are required for NH1-mediated immunity. Subsequent inoculation experiments confirmed that the suppression of the NH1-mediated lesion mimic phenotype correlated with enhanced susceptibility to the bacterial pathogen *Xoo*.

Previous reports have demonstrated the potential usefulness of comparative genome hybridization (CGH) or similar comparative genome techniques for plant species [17], [18], [19], [20], [21], [22], [23], however routine use of this technique in crop species remains limited. In this report we have successfully combined CGH and fine mapping with RNAi silencing and allelic complementation. In this way we were able to determine that the *snl6* mutant phenotype was the result of disruption of LOC_Os01g45200. In cloning *Snl6* we generated a population segregating for *NH1ox*, *Xa21* and *Snl6*. We were able to show that mutations in *Snl6* do not compromise resistance mediated by the rice pattern recognition receptor, *Xa21* driven by the ubiquitin promoter. These results are consistent with studies from Arabidopsis that have shown that the PRR, FLS2, does not require NPR1 to initiate an immune response [38]. Notably, in this population over expression of NH1 confers a similar level of resistance as XA21, highlighting the strength of NH1ox-mediated resistance as previously demonstrated [14].

*Snl6* is annotated as a cinnamoyl-CoA reductase (CCR)-like gene, however overall similarity to previously characterized CCRs is low. CCRs catalyze the first committed step of the monolignol biosynthetic pathway. The first identified CCR was from *Eucalyptus* [47] followed a year later by an orthologue from tobacco [48]. To date, several CCRs have been characterized though their exact role in lignin biosynthesis is still unclear. Studies from Arabidopsis and tobacco indicate that down regulation of CCR results in severe developmental phenotypes, including collapsed xylem cells and dwarfism, a decrease in total lignin along with a higher S/G ratio in the lignin polymer, and the appearance of feruloyl tyramines [40], [41], [42], [49], [50]. Among the CCR family, a large amount of sequence variability exists, which may contribute to the overall uncertainty about the exact role of CCR. In Arabidopsis, two CCRs, AtCCR1 and AtCCR2 have been compared and attributed a role in lignin biosynthesis during development or pathogen attack, respectively. While AtCCR2 is primarily involved in pathogen defense, knockdown experiments have shown that it can at least partially compensate for a downregulated AtCCR1.

To the best of our knowledge there has been only one report characterizing a CCR-like gene from rice. OsCCR1 was originally identified based on its *in vitro* interaction with OsRac1. OsRac1 is a GTPase, important for the defense response in rice. While expression of *OsCCR1* was induced by a sphingolipid elicitor in suspension cells, no mutant phenotype was observed [31]. Notably, *OsCCR1* is dissimilar to *Snl6*, sharing only 28% identity at the amino acid level.

Here we show that mutations in *Snl6* disrupt the NH1ox-mediated constitutive activation of PR genes, providing a mechanism for the role of *Snl6* in NH1-mediated resistance. In addition, as *Snl6* is annotated as a CCR-like gene, we hypothesized that *snl6* mutant lines may contain alterations in the phenylpropanoid biosynthetic pathway. Indeed, we show that disruption of *Snl6* leads to less lignin accumulation. Based on these data alone, it is unclear whether *Snl6* contributes specifically to NH1ox-mediated resistance or as part of a more general resistance response. However, because *Snl6* is highly co-expressed with two additional members of the phenylpropanoid biosynthetic pathway, PAL and 4CL, and because both these genes are induced in NH1 over expression plants, it seems likely that over expression of NH1 does induce lignin biosynthesis. As lignin is an important defense molecule, providing a physical barrier to pathogen entry into the plant, our results indicate that *Snl6* has dual roles in the resistance response: activation of PR genes and lignin biosynthesis.

Lignin contributes to plant structure as well as pathogen defense. Most previously described lignin mutants contain a severe phenotypic detriment [34], [40], [41], [42]. Our result, that *snl6* mutants contain decreased lignin, is particularly relevant to studies of bioenergy crops because *snl6* mutant lines do not display an obvious morphologic phenotype. In addition, we observed a greater than 15% increase in sugar extractability from *snl6* mutants as compared to controls. Our results indicate the presence of a previously uncharacterized group of CCR-like genes, alterations in which can alter lignin content without affecting development.

## Materials and Methods

### NH1ox Mutant Screen

NH1ox seeds were treated with fast neutron (FN) mutagenesis in three batches at 18, 20 and 22 Grays, respectively. This M_0_ population was grown at the UC Davis rice field and M_1_ seed from approximately 4,000 M_0_ individuals was then collected as 400 pools (10 per pool). Approximately fifteen M_1_ seed from each of the M_0_ lines were subsequently grown in the field (n = ∼60,000 M_1_ seed). The 60,000 M_1_ lines were sprayed with 10 mM BTH at 5 weeks and 7 weeks and lesion mimic severity was assessed at 10 weeks. BTH treatment of NH1ox plants in the field resulted in a lesion mimic phenotype. Plants that did not develop lesions were called, Suppressor of NH1-mediated Lesion mimic (*snl*) mutants. *snl6-FN* (originally named line 11-3) was identified in this screen.

### *Xoo* Inoculations

*Xoo* (Philippine race 6, PXO99AZ) inoculations were carried out as previously described [25]. Briefly, 8-week old rice plants were transferred to the growth chamber. Scissors were dipped in a solution of *Xoo* cells in water (OD_600_ = 0.5) and then used to clip the rice leaf tip. Growth curves were performed as previously described [36].

### Comparative Genome Hybridization

In collaboration with Nimblegen, we designed a full genome tiling array for rice, *ssp*. Japonica (average one 50-mer probe/146bp). CGH was conduced following Nimblegen's specified procedures. Briefly, genomic DNA from *snl6-FN* and NH1ox plants was isolated and the quality of the DNA was assessed via a spectrophotometer and agarose gel electrophoresis prior to sending to Nimblegen. At Nimblegen, the genomic DNA was sheared, labeled and then hybridized to the tilling array. A negative log_2_ ratio represents a deletion in the genome of *snl6-FN*.

### Creation of Line BB1-1

*snl6-FN* (line: 11-3-2) was crossed to a transgenic line expressing, *Xa21* (*Ubi Myc-Xa21* (line 7A-8), (Park, Ronald submitted) pollen donor) under control of the ubiquitin promoter, in a kitaake background. The cross was confirmed by testing the F1 progeny (line: BB1) for the presence of *Xa21.* The F1 progeny was allowed to self-pollinate to create an F2 population (line: BB1-1) that segregated for *Xa21*, *NH1ox*, and all 5 deletions identified from CGH.

#### Fine mapping

Individuals from line BB1-1 (above) were grown in the greenhouse and genotyped for the presence of *Xa21*, *NH1ox*, and all 5 deletions identified from CGH. Individuals that did not contain *Xa21* (as *Xa21* also confers resistance to *Xoo*, *Xa21*+ individuals were excluded from this experiment) were moved to the growth chamber for challenge with *Xoo* and lesion lengths were assessed after 14 days. One individual (line: BB1-1-8A) contained *NH1ox*, was susceptible to *Xoo*, and showed a recombination event between the two deletions on chromosome 1. Phenotype and genotype were assessed in the next generation (line BB1-1-8A).

#### *Xa21* effect on *Snl6*

Individual progeny from line BB1-1 (described above) were grown in the greenhouse and genotyped for the presence of *Xa21*, *NH1ox*, and *Snl6*. Plants were moved to the growth chamber for challenge with *Xoo* and lesion lengths were assessed after 14 days. Primer sequences listed in Table S3.

### Creation of *snl6-RNAi*

A silencing construct for LOC_Os01g45200 (Genbank: Os01g0639200) was created following our previously described strategy [36]. PCR was used to amplify 425 bp from the 3′ end of LOC_Os01g45200 (Primers: GAATTCAGGCTTCGATACGAGCATGT; AGATCTGTCGAATGCGACGGAGTAG). This PCR product was confirmed through sequencing and cloned in inverse orientation (separated by ∼1000 bp of GUS intron sequence) into a gateway compatible pENTR (Invitrogen) vector. The inverted repeat sequence was then recombined into a modified version of the binary vector Ubi-pC4300 encoding a gene for mannose selection.

### *snl6-RGT* Allele Identification

*snl6-RGT* (RGT6140B_5.1) was identified from the Rice Transposon Flanking Sequence Tag (FST) Database: http://sundarlab.ucdavis.edu/rice/blast/blast.html. *snl6-RGT* was crossed with *snl6-FN* (pollen donor) and the cross was confirmed through PCR with primers specific to the NH1ox transgene (not present in *snl6-RGT*).

### Realtime Quantitative RT-PCR Analysis

Realtime reactions were prepared using Bio-RAD SsoFast EvaGreen Supermix and run on a BIO-RAD CFX96 Real-time System. Primers were designed using the Beacon Designer program and R^2^ and efficiency were determined for each primer pair (Tables S2 and S4). When gene expression from a single plant is displayed, error bars represent standard deviation of 3 technical replicates. When multiple plants are combined, data represent biological replicates (each with 3 technical replicates) for each genotype and the s.e.m and number of individuals (n) is reported.

### Phloroglucinol Staining

Leaf tissue was collected from plants just before the emergence of the panicle and cleared in a solution of ethanol, lactic acid and phenol (2∶1∶1) as previously described [27]. Cleared tissue was then soaked in 0.6% Phloroglucinol (in 2∶1 ethanol/HCL). Tissue was vacuum infiltrated for one hour and then left in the dark over night. Note: the reported phenotype is dependent on the tissue type, age and health of the plants. Altered phenolic content in *snl6* mutant plants was not observed in roots or stems, in plants showing high levels of stress or in young plants.

### Sugar Analysis

Alcohol insoluble residue (AIR) was prepared from ground mature rice leaves and destarched with alpha-amylase (Megazyme). All analyses were done using five mg of destarched AIR. For hemicellulose sugar composition, AIR was treated with 2M TFA at 120C for 1 h, dried and re-suspended in water; an aliquot was taken for analysis by high performance anion exchange chromatography with pulse amperometry detection (HPAEC-PAD) using a Carbopac PA20 analytical column (Dionex) and monosaccharide standards. Cellulose content was estimated as glucose equivalents using HPAEC-PAD from AIR treated with sulfuric acid (72% w/w) for 1 h at 30C, then diluted to 4% and incubated at 120C for 1 h. For enzymatic saccharification, AIR was first pre-treated in water at 100C for 1 h, then a mixture of cellulase and beta-glucosidase (5% w/w dose, Novozyme) in 0.1 M citrate buffer, pH 5.0 was added (reaction volume of 1% total solids); samples were incubated at 50C for 8 h and an aliquot was then taken to calculate total reducing sugar amounts using the DNS assay.

### Sequence Analysis

Protein sequences were obtained from NCBI, TIGR or the JGI Brachypodium resource and compared using the web-based ClustalW software (http://www.ebi.ac.uk/Tools/clustalw2/index.html).

## Supporting Information

Figure S1 *snl6-FN* over-expresses NH1. Quantitative RT-PCR was used to determine the relative amounts of NH1 expression in LG, NH1ox and *snl6-FN* (line: 11-3-2-1) plants. Mean ± s.e.m, n = 3.
(0.21 MB TIF)

Figure S2 Annotated genes in Deletion 1B and schematic of *snl6* RNAi and insertion lines. (Top) CGH results for Deletion 1B showing predicted gene models. Gene annotation is based on TIGR v 5. (Bottom) Schematic of *snl6-RNAi* and *snl6-RGT*. An inverted repeat RNAi construct was created to silence Os01g45200. PCR was used to amplify the 3′ end of Os01g45200 and the resulting fragment was cloned in inverse orientation, separated by an approximately 1 kb spacer. To identify the location of the insertion in *snl6-RGT* (line: RGT6140B_5.1), insertion specific primers (Ds5'-2a) were combined with primers specific to Os01g45200. The resulting PCR product was sequenced and revealed that the insertion is in the first intron of Os01g45200.
(0.64 MB TIF)

Figure S3 Four *snl6-RNAi* T0 lines are susceptible to *Xoo*. Eight-week old independently transformed lines were challenged with *Xoo*. Lesion length development after 12 days. Mean ± s.d., n = 3 are displayed.
(0.22 MB TIF)

Figure S4 Protein alignment for *Snl6* and its homologues from other species. Close homologs were collected from NCBI (maize: ACR34585.1, Sorghum: EES03334.1), The Brachypodium Sequence Resource (JGI) (2g44800.1), TAIR (Arabidopsis: AT5G14700) and TIGR (rice: Os05g50250). Previously described CCRs were found through literature searches and sequences collected in NCBI as follows: Arabidopsis (AtCCR1: NP_173047, AtCCR2: NP_178197), poplar (PtCCR: AJ224986), switchgrass (PvCCR1a: GQ450296 PvCCR2a: GQ450301), and rice (OsCCR1). All sequences were compared using ClustalW2 web-based software.
(0.52 MB TIF)

Figure S5 *Snl6* mutants display no obvious developmental defects. Plant height and tiller number were evaluated at 8 weeks and a picture was taken of each genotype. Seed set was evaluated (total panicle weight) after senescence. LG, NH1ox and *snl6-FN* (line 11-3-2-1) are compared. n>10; s.d. from the mean.
(0.77 MB TIF)

Figure S6 Sugar analysis reveals higher sugar release from *snl6* mutant lines. Cell wall extraction (AIR) was performed on LG, NH1ox and *snl6-FN* (lines 11-3-2 or 11-3-4) adult rice leaves. Overall cell wall sugar content (A) and relative sugar release (enzymatic saccharification) after hot water pre-treatment (B), ± s.e.m. n = 3. Letters (a or b) indicate significant difference (p<0.05).
(0.38 MB TIF)

Table S1 Complete list of deleted genes in *snl6-FN*.
(0.06 MB PDF)

Table S2 Efficiency (E) and R2 values for realtime PCR primers.
(0.06 MB PDF)

Table S3 Genotyping primers.
(0.06 MB PDF)

Table S4 Realtime PCR primers.
(0.05 MB PDF)
