# Supplementary figures and images for: Rice Snl6, a Cinnamoyl-CoA Reductase-Like Gene Family Member, Is Required for NH1-Mediated Immunity to Xanthomonas oryzae pv. oryzae
Source: PLoS Genet. 2010 Sep 16;6(9):e1001123. doi: 10.1371/journal.pgen.1001123 (PMC2940737; doi:10.1371/journal.pgen.1001123)

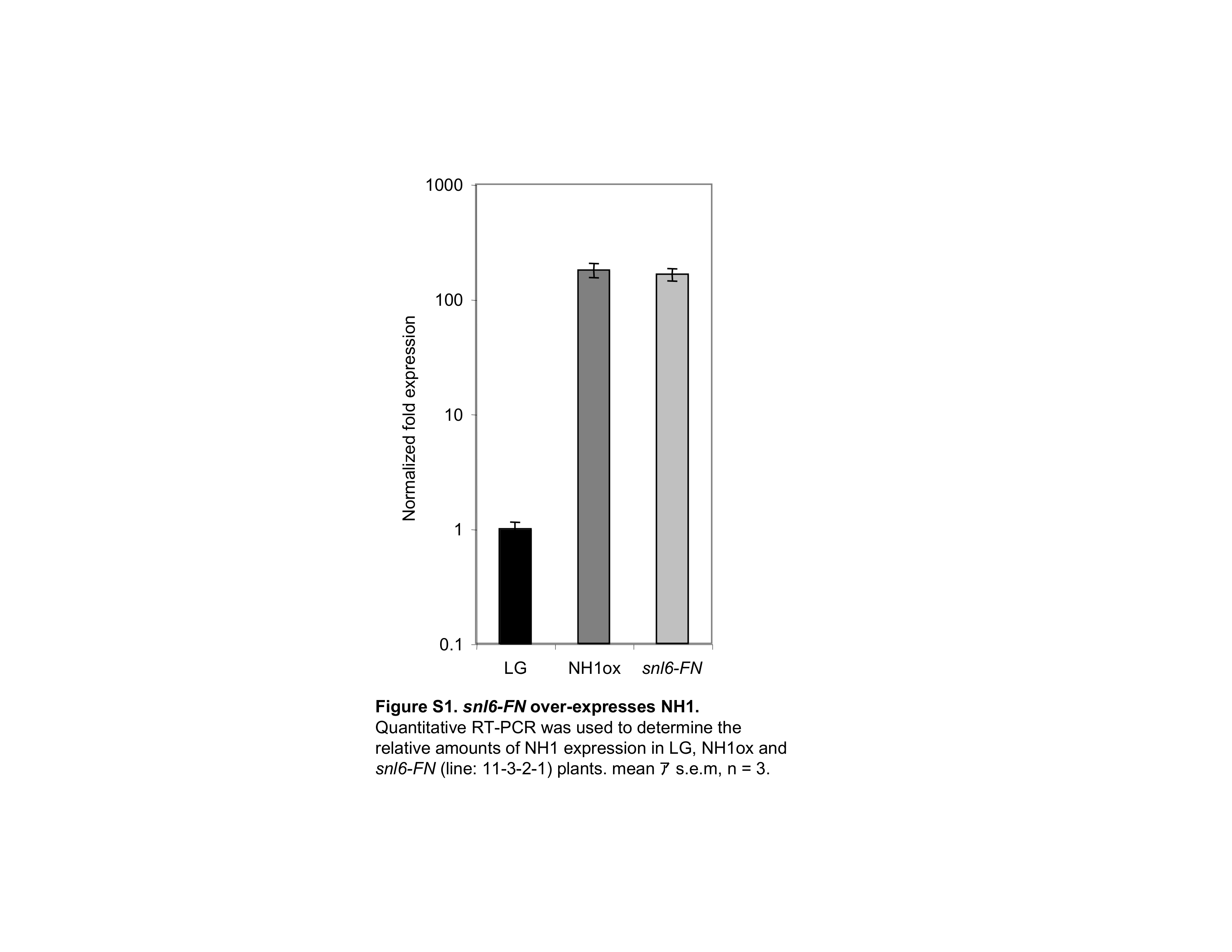

Supplement: Figure S1 — snl6-FN over-expresses NH1. Quantitative RT-PCR was used to determine the relative amounts of NH1 expression in LG, NH1ox and snl6-FN (line: 11-3-2-1) plants. Mean ± s.e.m, n = 3. (0.21 MB TIF) [file pgen.1001123.s001.tif]

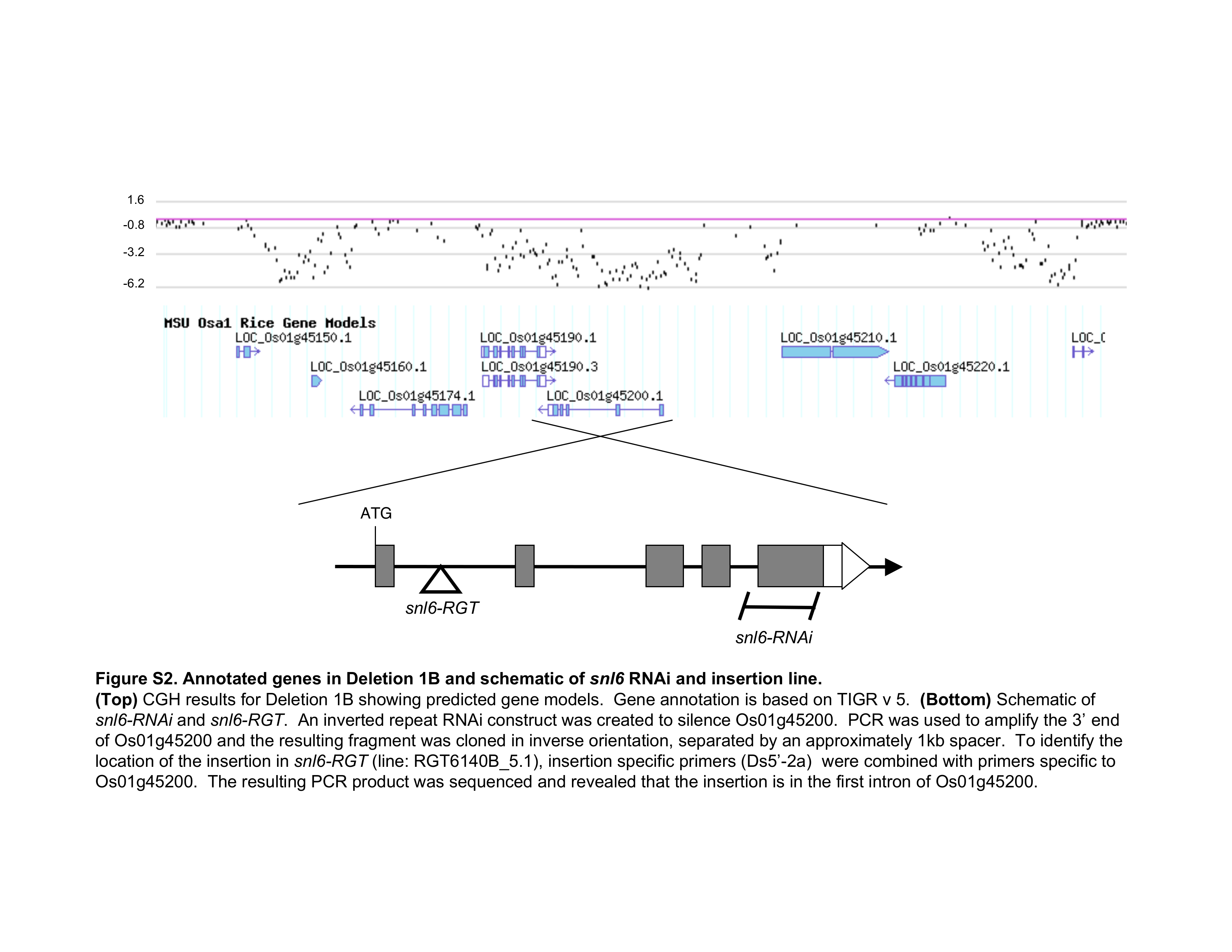

Supplement: Figure S2 — Annotated genes in Deletion 1B and schematic of snl6 RNAi and insertion lines. (Top) CGH results for Deletion 1B showing predicted gene models. Gene annotation is based on TIGR v 5. (Bottom) Schematic of snl6-RNAi and snl6-RGT. An inverted repeat RNAi construct was created to silence Os01g45200. PCR was used to amplify the 3′ end of Os01g45200 and the resulting fragment was cloned in inverse orientation, separated by an approximately 1 kb spacer. To identify the location of the insertion in snl6-RGT (line: RGT6140B_5.1), insertion specific primers (Ds5'-2a) were combined with primers specific to Os01g45200. The resulting PCR product was sequenced and revealed that the insertion is in the first intron of Os01g45200. (0.64 MB TIF) [file pgen.1001123.s002.tif]

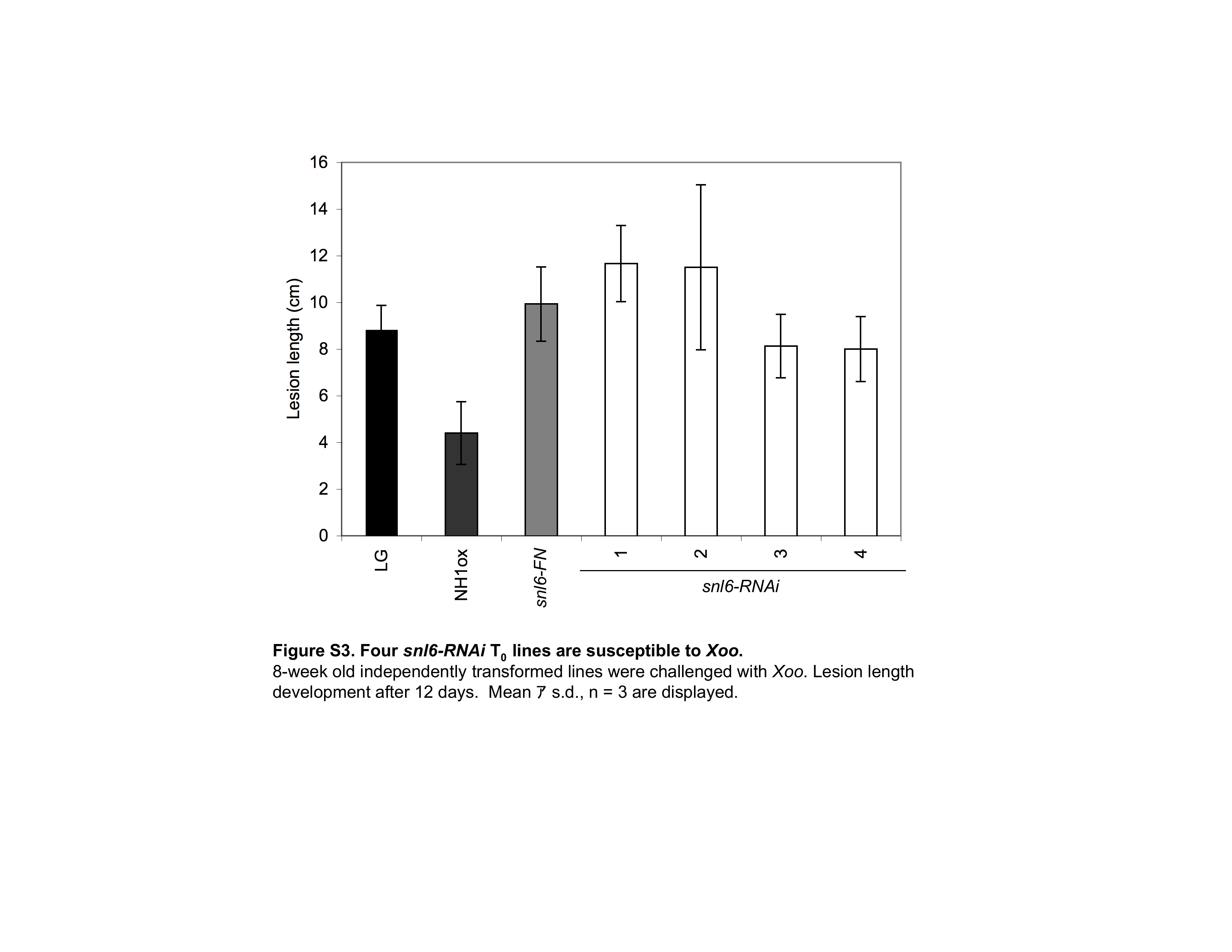

Supplement: Figure S3 — Four snl6-RNAi T0 lines are susceptible to Xoo. Eight-week old independently transformed lines were challenged with Xoo. Lesion length development after 12 days. Mean ± s.d., n = 3 are displayed. (0.22 MB TIF) [file pgen.1001123.s003.tif]

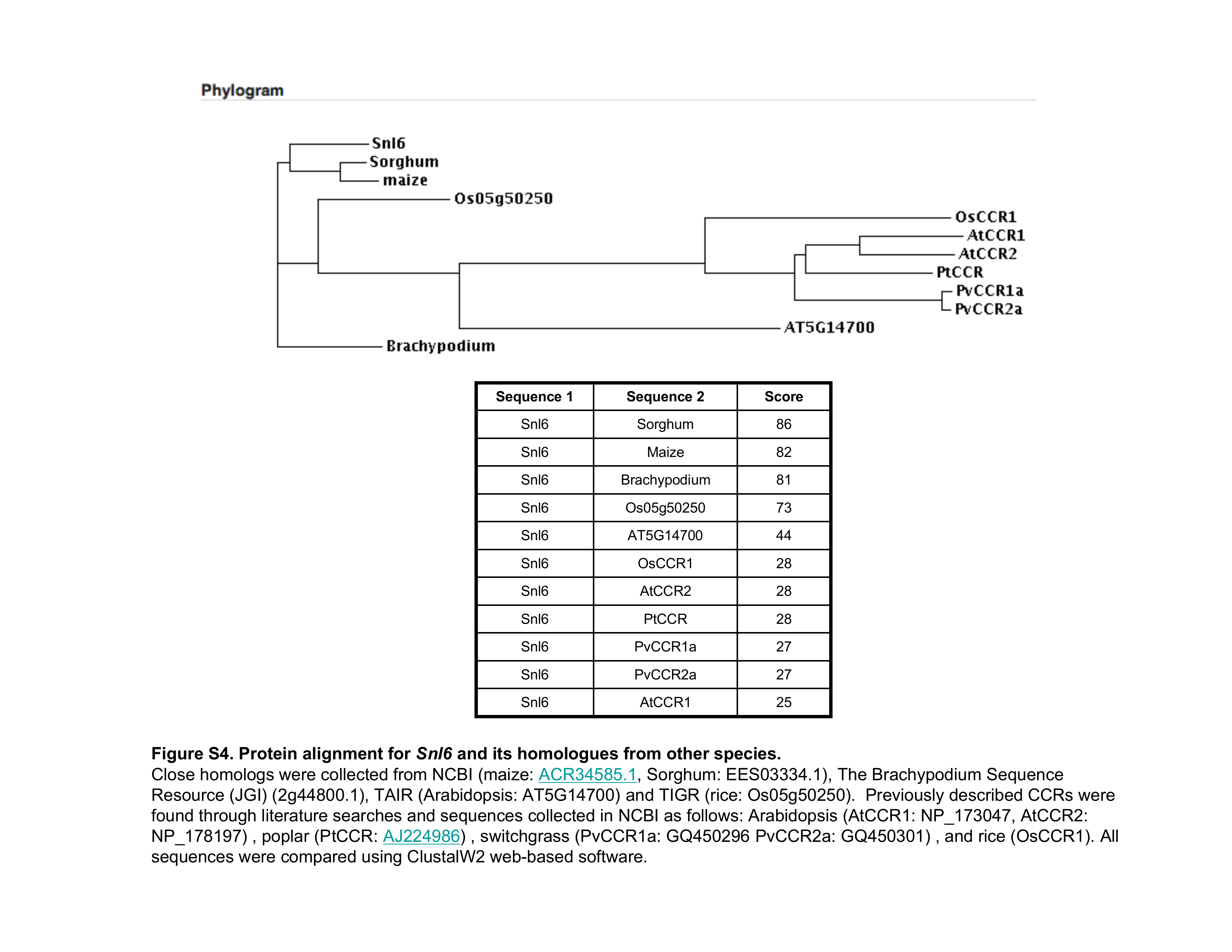

Supplement: Figure S4 — Protein alignment for Snl6 and its homologues from other species. Close homologs were collected from NCBI (maize: ACR34585.1, Sorghum: EES03334.1), The Brachypodium Sequence Resource (JGI) (2g44800.1), TAIR (Arabidopsis: AT5G14700) and TIGR (rice: Os05g50250). Previously described CCRs were found through literature searches and sequences collected in NCBI as follows: Arabidopsis (AtCCR1: NP_173047, AtCCR2: NP_178197), poplar (PtCCR: AJ224986), switchgrass (PvCCR1a: GQ450296 PvCCR2a: GQ450301), and rice (OsCCR1). All sequences were compared using ClustalW2 web-based software. (0.52 MB TIF) [file pgen.1001123.s004.tif]

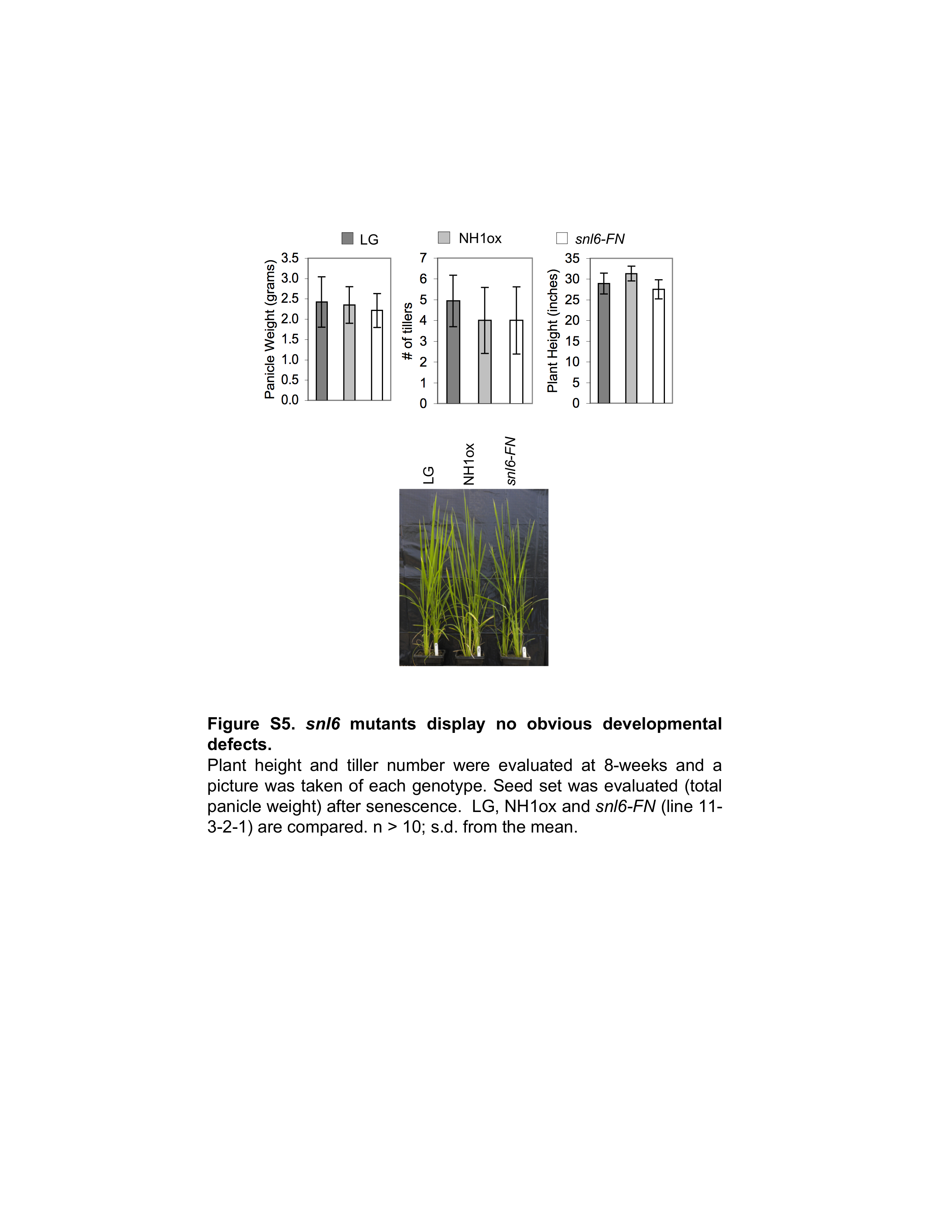

Supplement: Figure S5 — Snl6 mutants display no obvious developmental defects. Plant height and tiller number were evaluated at 8 weeks and a picture was taken of each genotype. Seed set was evaluated (total panicle weight) after senescence. LG, NH1ox and snl6-FN (line 11-3-2-1) are compared. n>10; s.d. from the mean. (0.77 MB TIF) [file pgen.1001123.s005.tif]

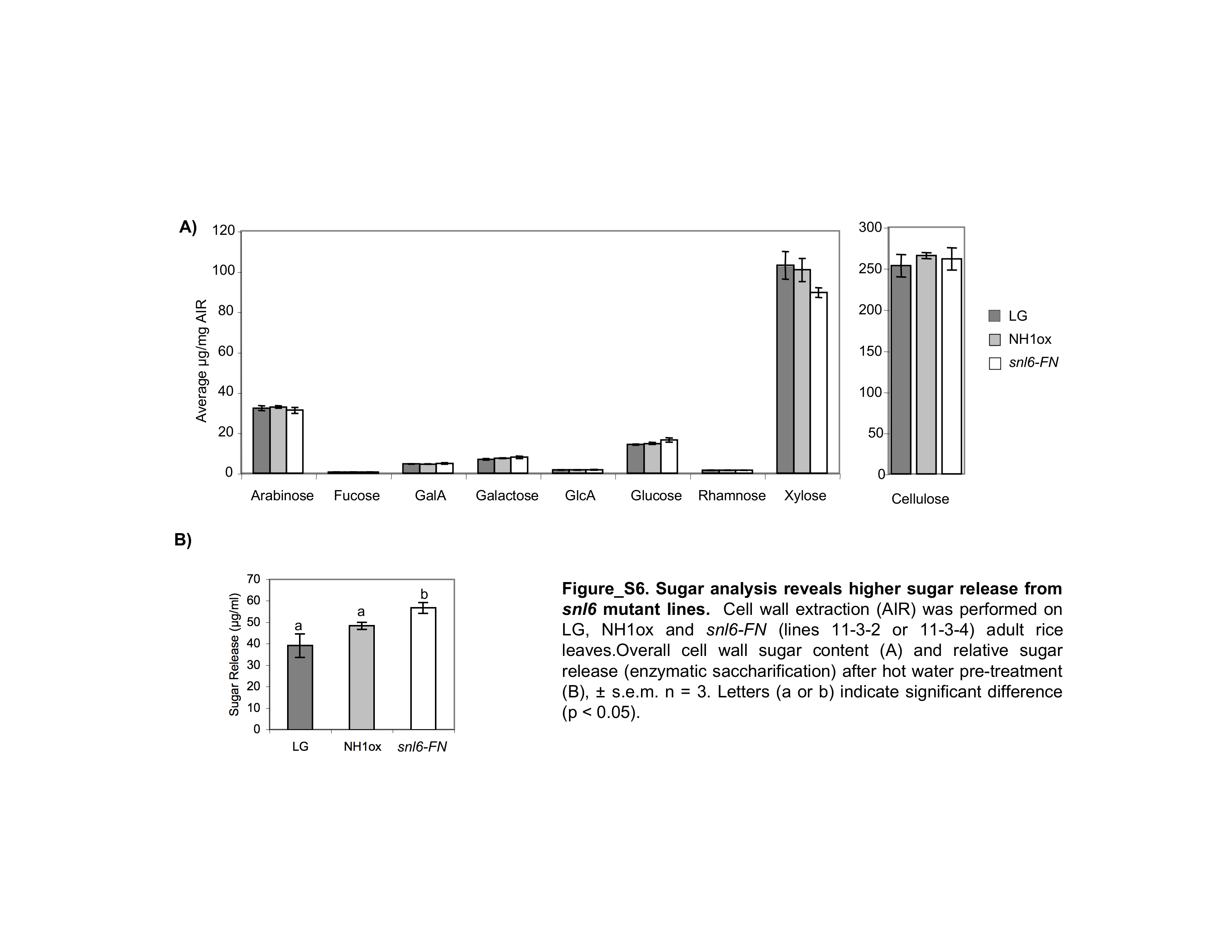

Supplement: Figure S6 — Sugar analysis reveals higher sugar release from snl6 mutant lines. Cell wall extraction (AIR) was performed on LG, NH1ox and snl6-FN (lines 11-3-2 or 11-3-4) adult rice leaves. Overall cell wall sugar content (A) and relative sugar release (enzymatic saccharification) after hot water pre-treatment (B), ± s.e.m. n = 3. Letters (a or b) indicate significant difference (p<0.05). (0.38 MB TIF) [file pgen.1001123.s006.tif]
