# Supplementary material for: Rice Snl6, a Cinnamoyl-CoA Reductase-Like Gene Family Member, Is Required for NH1-Mediated Immunity to Xanthomonas oryzae pv. oryzae
Source: PLoS Genet. 2010 Sep 16;6(9):e1001123. doi: 10.1371/journal.pgen.1001123 (PMC2940737; doi:10.1371/journal.pgen.1001123)
Supplement: Table S1 — Complete list of deleted genes in snl6-FN. (0.06 MB PDF) [file pgen.1001123.s007.pdf]

Table S1. Complete list of deleted genes in *snl6-FN.*

	Locus ID	TIGR Annotation
Deletion 1A (57 kbp)	Os01g21370	Transposon protein, putative
	Os01g21380	FAD dependent oxidoreductase domain containing protein, expressed
	Os01g21390	Hypothetical Protein
	Os01g21400	Retrotransposon putative
	Os01g21410	Expressed Protein
	Os01g21420	pre-mRNA-splicing factor SF2, expressed
	Os01g21430	Retrotransposon protein, putative, Ty3-gypsy subclass
	Os01g21440	Expressed Protein
	Os01g21450	Expressed Protein
	Os01g21480	Transposon protein, putative, CACTA, En/Spm sub-class
Deletion 1B (13.5 kbp)	Os01g45160	Conserved hypothetical protein
	Os01g45174	transposon protein, putative
	Os01g45190	DEAD-box ATP-dependent RNA helicase, putative, expressed
	Os01g45200	Cinnamoyl-CoA reductase-related, putative, expressed
	Os01g45210	Retrotransposon protein, putative, Ty3-gypsy subclass
	Os01g45220	Retrotransposon protein, putative, Ty3-gypsy subclass
Deletion 2 (3 kbp)	Os02g33730	Ubiquinol-cytochrome C reductase hinge protein, putative, expressed
Deletion 3 (100.5 kbp)	Os03g56070	Expressed Protein
	Os03g56080	Hypothetical Protein
	Os03g56090	MYB family transcription factor, putative, expressed
	Os03g56100	hypothetical protein
	Os03g56110	Homeobox protein knotted-1, putative, expressed
	Os03g56120	Hypothetical Protein
	Os03g56130	Lichenase-2 precursor, putative
	Os03g56140	Homeobox protein rough sheath 1, putative expressed
	Os03g56149	Hypothetical Protein
	Os03g56160	Lectin-like receptor kinase 7, putative, expressed
	Os03g56170	Conserved hypothetical protein
	Os03g56180	Legume lectins beta domain containing protein, expressed
	Os03g56190	Cytochrome c oxidase-related, putative, expressed
	Os03g56200	Expressed Protein
	Os03g56220	Stress-induced protein, putative, expressed
	Os03g56234	myb/SANT domain protein, putative, expressed
	Os03g56241	40S ribosomal protein S29, putative, expressed
Deletion (6 kbp)	Os07g35810	TKL_IRAK_DUF26-ld.6 - kinase, expressed
