# Supplementary material for: Rice Snl6, a Cinnamoyl-CoA Reductase-Like Gene Family Member, Is Required for NH1-Mediated Immunity to Xanthomonas oryzae pv. oryzae
Source: PLoS Genet. 2010 Sep 16;6(9):e1001123. doi: 10.1371/journal.pgen.1001123 (PMC2940737; doi:10.1371/journal.pgen.1001123)
Supplement: Table S2 — Efficiency (E) and R2 values for realtime PCR primers. (0.06 MB PDF) [file pgen.1001123.s008.pdf]

Table S2. Efficiency (E) and R^2 values for realtime PCR primers.

**Primers**	**E (%)**	**R^2**
01g45190	99.9	0.961
01g45200 (*Snl6*)	104.7	0.989
03g18850 (PR10-famil y)	9 9 . 1	0.984
12g36850 (PR10-family)	98.3	0.986
02g41650 (PAL-family)	107.8	0.900
NH1	91.2	0.985
UBQ5	105.5	0.981
