# Supplementary material for: Rice Snl6, a Cinnamoyl-CoA Reductase-Like Gene Family Member, Is Required for NH1-Mediated Immunity to Xanthomonas oryzae pv. oryzae
Source: PLoS Genet. 2010 Sep 16;6(9):e1001123. doi: 10.1371/journal.pgen.1001123 (PMC2940737; doi:10.1371/journal.pgen.1001123)
Supplement: Table S3 — Genotyping primers. (0.06 MB PDF) [file pgen.1001123.s009.pdf]

Table S3. Genotyping primers.

Gene	Forward	Reverse
Xa21	ATAGCAACTGATTGCTTGG (H3 frag F)	CGATCGGTATAACAGCAAAAC (3' Xa21 R)
NH1ox	TGATATACTTGGATGATGGCA (Ubi-1)	GGACGGCGATGCGCGCGTC (PrNH22)
Deletion 1A	AGCAGGAGGGAGAAGACACA (Os01g21420_F)	ACCAGGAAGCAAAGCAAAGA (Os01g21420_R)
Deletion 1B	GAAGACGAACCGCGTCTG (Os01g45160_F)	GGTGTCACCTCTGATCACCTG (Os01g45160_R)
Deletion 2	GTTGCTTGTTGGTGGGAACT (Os02g33730_F)	TCCGTGGGAAAATCAAAGAC (Os02g33730_R)
Deletion 3	TCCTCAGCAGCTTACCACCT (Os03g56234_F)	TGCGTCGAACAAGACGATAG (Os03g56234_R)
Deletion 7	TACACCGGTAATCCGATGGT (Os07g35810_F)	GGCCCAAGGACTAAGGTCTC (Os07g35810_R)
