# Supplementary material for: Rice Snl6, a Cinnamoyl-CoA Reductase-Like Gene Family Member, Is Required for NH1-Mediated Immunity to Xanthomonas oryzae pv. oryzae
Source: PLoS Genet. 2010 Sep 16;6(9):e1001123. doi: 10.1371/journal.pgen.1001123 (PMC2940737; doi:10.1371/journal.pgen.1001123)
Supplement: Table S4 — Realtime PCR primers. (0.05 MB PDF) [file pgen.1001123.s010.pdf]

Table S4. Realtime PCR primers.

Gene	Forward	Reverse
NH1	CAGGTGAGAGTCTACGAGGAAGG (NH1_RT_S2)	TTGTCTTTCAGGAGGTGGATTTGC (NH1_RT_AS2)
Os01g45190	GCTATCGGTGACTTCATCAATATC (BB190_RT_S1)	AGACTCTGCCAGGTGTTCC (BB190_RT_AS1)
Os01g45200	CACCGTGGACGGTGATGGCGAACGTG (200_RNAi_F2)	GCAACGACGACGTGAAGACGC (200_cDNA_R2)
03g1885 0	AGTTCCTGGACGTGGACAAG	TCTCGTCCTTCACCTCCACT
12g3685 0	ACGCAGGGAGCGTATACAAG	CACCCTGCTCTTAACCTCCA
02g4165 0	GGCCTCCACATCGCTCGC	ACGGCCTCGCGGTCGA
Ubiquitin	ATGGCCAACCACTTCGACCG (UBQ5_F)	TAAGCCTGCTGGTTGTAGACGT (UBQ5_R)
